# Estimates of the changing age-burden of *Plasmodium falciparum* malaria disease in sub-Saharan Africa

**DOI:** 10.1038/ncomms4136

**Published:** 2014-02-11

**Authors:** Jamie T. Griffin, Neil M. Ferguson, Azra C. Ghani

**Affiliations:** 1 MRC Centre for Outbreak Analysis and Modelling, Department of Infectious Disease Epidemiology, Imperial College London, London W2 1PG, UK

## Abstract

Estimating the changing burden of malaria disease remains difficult owing to limitations in health reporting systems. Here, we use a transmission model incorporating acquisition and loss of immunity to capture age-specific patterns of disease at different transmission intensities. The model is fitted to age-stratified data from 23 sites in Africa, and we then produce maps and estimates of disease burden. We estimate that in 2010 there were 252 (95% credible interval: 171–353) million cases of malaria in sub-Saharan Africa that active case finding would detect. However, only 34% (12–86%) of these cases would be observed through passive case detection. We estimate that the proportion of all cases of clinical malaria that are in under-fives varies from above 60% at high transmission to below 20% at low transmission. The focus of some interventions towards young children may need to be reconsidered, and should be informed by the current local transmission intensity.

## Abstract (web summary)

Reduction in malaria transmission has changed the age pattern of malaria incidence. This study brings insights into the changes in age distributions of clinical malaria across Africa, with importance for improving within-population targeting of malaria control interventions.

## Graphical abstract

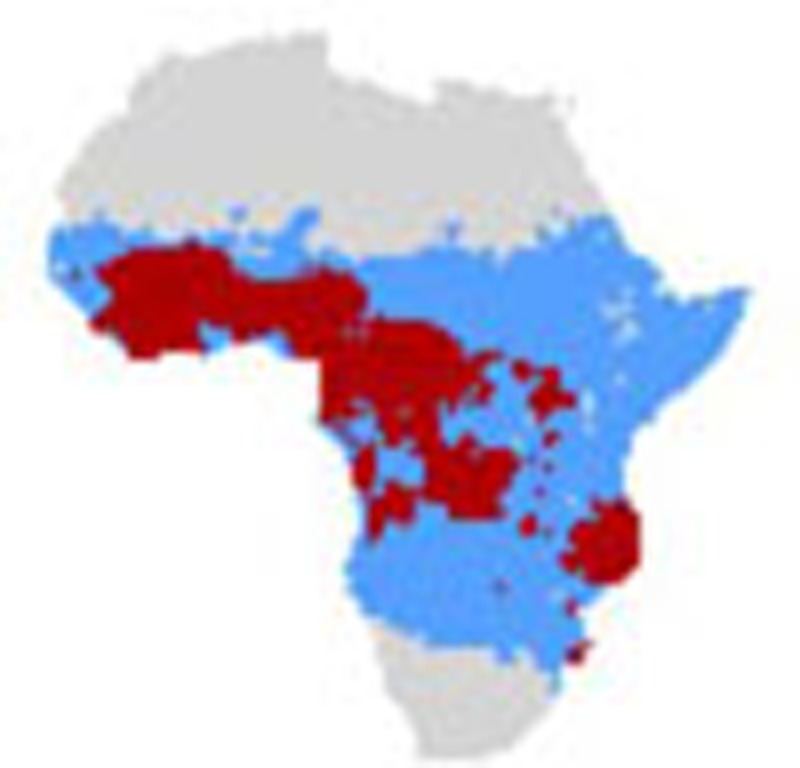

In recent years, the burden of disease due to malaria has fallen in many parts of sub-Saharan Africa, often coinciding with the introduction of more effective treatments and the scale-up of long-lasting insecticide-treated net ownership and use^1^. Patterns of clinical disease vary by age and transmission intensity: in highly endemic areas, the disease burden is greatest in infants and young children, while in areas of lower transmission many cases also occur in older children and adults^2^. With declining transmission, there have been shifts in cases to older ages. For example, in south-western Senegal, a 30-fold drop in malaria incidence between 1996 and 2010 was accompanied by a shift in the age distribution of cases, with 34% of cases in the under-fives in 1996 falling to ~5% in 2010 (ref. 3). Similarly, in western Gambia, a rapid fall in the proportion of malaria admissions between 2003 and 2007 was accompanied by an increase in the mean age of paediatric malaria admissions from 3.9 years to 5.6 years^4^. This changing distribution of cases is most likely due to the slower development of naturally acquired immunity. Understanding and predicting these changes is important to ongoing control. As the case distribution shifts towards older ages, there is a need to re-assess age-targeted control to ensure that those at the highest risk of developing clinical disease and its sequelae are sufficiently protected.

Monitoring changes in disease burden remains difficult, however. Not all cases seek care at public clinics^5^, and in many countries with the highest burden, health reporting systems are insufficient to accurately capture trends. Even in research studies, a variety of approaches have been adopted to assess burden. Active case detection (ACD) is the most sensitive but expensive approach, where households are visited at a predefined frequency, asked to report any fevers and blood tests are used to identify high parasite densities. Other studies rely on passive case detection (PCD) via local health clinics. It is generally acknowledged that the former gives a more accurate picture of the true burden of disease, although with frequent intervention and treatment, ACD may also modify transmission patterns^6^.

Given the limitations of disease reporting, alternative approaches to estimate disease burden have been sought. Current WHO methodology uses an estimate of the disease incidence by age group and endemicity level to obtain estimates of disease burden in countries in which the health reporting system is considered insufficient to rely on clinic-based case reports^7^. Alternative disease burden estimates have been obtained by using an empirical statistical model to capture the relationship between disease incidence and parasite prevalence and combine this with spatially stratified prevalence estimates into disease burden estimates^8^. One advantage of the latter approach is that it uses the rich set of parasite prevalence estimates that are available rather than more limited data on clinical disease, and in addition, it can potentially be used to assess the changing disease burden over time. However, the relationship between parasite prevalence and disease incidence was obtained for the population as a whole, and so the method does not capture changing patterns in the age distribution of cases. A third approach has been adopted to capture the shifting age distribution using mathematical models^9^. However, that study only considered three data sets from Senegal and Tanzania, meaning the extent to which the results can be extrapolated to other endemic settings is unclear.

Here we fit a mathematical model of malaria transmission to data from a wide range of studies in Africa, which report parasite prevalence and disease incidence. The model explicitly includes key aspects of malaria biology, notably the acquisition and loss of immunity, and therefore can capture the observed dependence of the age distribution of cases on transmission intensity. Furthermore, we stratify our analysis by case detection methods (namely ACD and PCD). We then combine our results with spatially stratified estimates of parasite prevalence to obtain age-stratified disease burden estimates for sub-Saharan Africa, which can be compared with both previous ACD-derived estimates and those derived using PCD. More generally, our method provides a framework for assessing the changing age distribution of cases and so can be used with any underlying estimates of parasite prevalence at a range of spatial scales to improve estimates of the population at the highest risk from malaria, and hence better inform age-targeted control.

## Results

### Data

**Figure 1:**
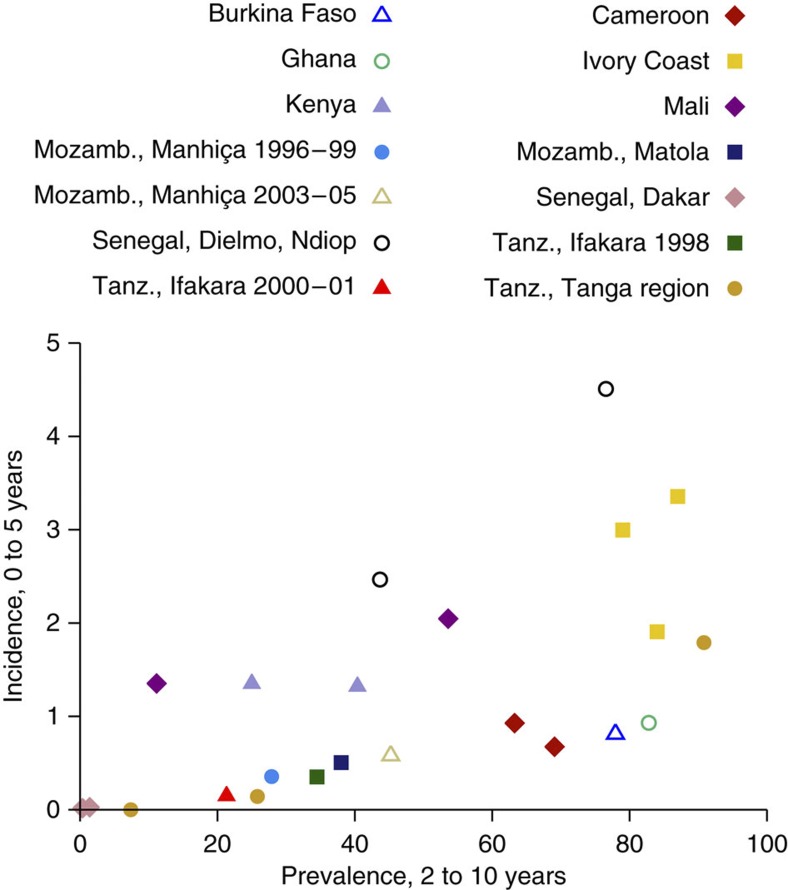
Incidence of clinical malaria in 0–5 year olds plotted against prevalence. Solid symbols indicate studies in which both clinical incidence and parasite prevalence were reported; hollow symbols indicate studies in which only clinical incidence is reported and parasite prevalence was estimated from alternative data (based on the prior EIR). The legend states the country, and also the place and year(s) where necessary to identify the study. Units for incidence are episodes per person per year.

Data were extracted from 23 sites reported in 14 studies on the relationship between parasite prevalence or entomological inoculation rate (EIR) and the incidence of disease: the studies are summarized in Table 1. Eleven sites had a contemporary EIR estimate. A summary of the estimates of clinical incidence in 0–5 year olds is plotted against microscopy-determined prevalence in 2–10 year olds in Fig. 1. As previously reported, there is wide variation in clinical incidence estimates at similar underlying levels of transmission^10^. For example, in areas where parasite prevalence in 2–10 year olds is measured at ~20%, the reported clinical incidence varies from 0.15 to 1.35 episodes per child per year while in areas with prevalence above 60%, it varies from 0.68 to 4.5 episodes per year. Case-reporting methods capture some of the variation, with higher rates of disease incidence reported via ACD compared with PCD.

### Fitted model

**Figure 2:**
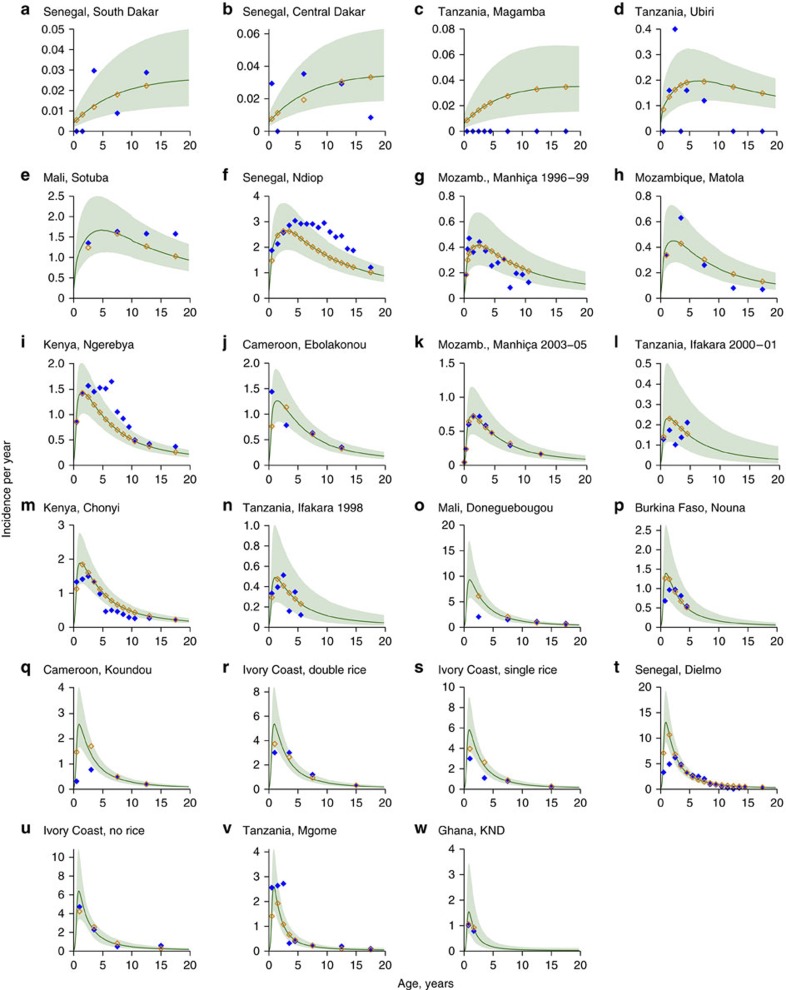
Observed and fitted incidence of clinical malaria by age. (**a**–**w**) Model fits are the median and 95% interval of the predicted incidence over the joint posterior distribution of the parameters and study-specific random effects. Sites are labelled by country and place, with the study year(s) to distinguish studies in the same place. Plots are ordered by posterior EIR. The blue solid symbols are the observed data; the green lines and shaded areas are the fitted model with 95% credible intervals; and the hollow orange symbols are the fitted model averaged over the age groups in the observed data.

We fitted an extended version of a previously published transmission model to the data^11^. The fit of the model to the individual studies is shown in Fig. 2. The model captures the peak of disease incidence in young children observed in some high transmission settings (Fig. 2r–u) as well as the widening of the distribution of cases to include older ages at lower transmission (Fig. 2g–n), although the magnitude and location of the peak is sometimes missed. While some of the variation between studies in similar transmission areas is explained by reporting, a substantial study-level random effect (coefficient of variation of 1.17) was also estimated reflecting an unexplained variation in the data. Some variation beyond what the model predicts in the shape of the curve of incidence against age was observed. For example, the data in Fig. 2f show a markedly different shape from other moderate transmission settings, with high incidence extending to 15 years of age.

**Figure 3:**
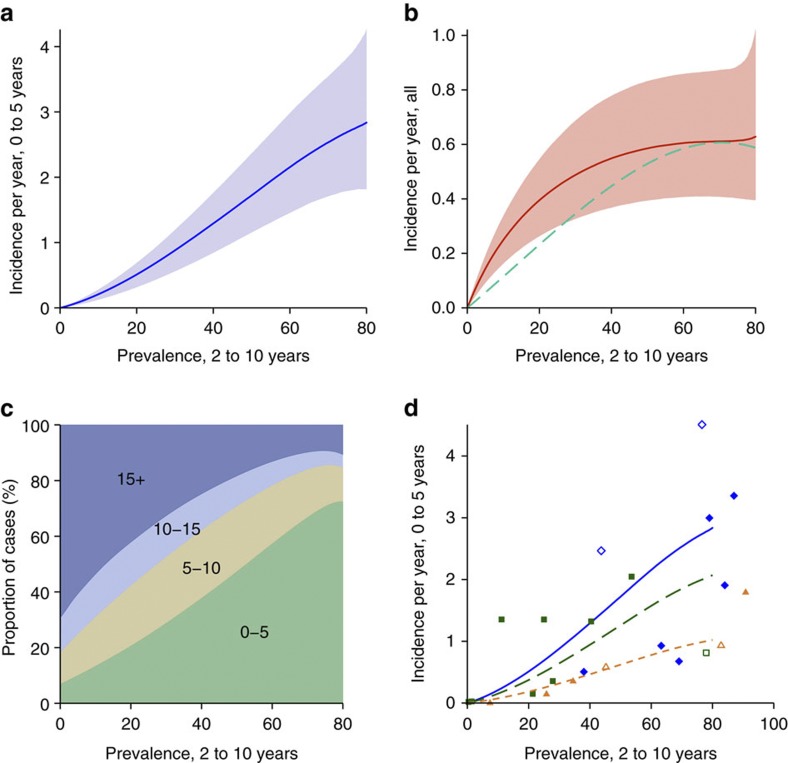
Fitted incidence and age distribution of cases plotted against prevalence. (**a**) The estimated relationship between parasite prevalence in 2–10 year olds and clinical incidence of disease in 0–5 years olds. The shaded areas represent the 95% credible intervals. (**b**) The estimated relationship between parasite prevalence in 2–10 year olds and overall clinical incidence (red solid line). The green dashed line shows the relationship estimated in Patil *et al*.^10^ (**c**) The shifting age-burden of disease at different levels of endemicity. The figure shows the estimated proportion of cases in each age group plotted against prevalence. (**d**) The estimated relationship between prevalence and clinical disease incidence in 0–5 year olds by detection method: blue—daily ACD; dark green—weekly ACD; orange—PCD. Lines are model predictions; solid symbols are observed incidence and prevalence; hollow symbols are observed incidence and model-fitted prevalence (based on the prior EIR).

The fitted model predicts a monotonically increasing clinical case incidence with prevalence (Fig. 3a,b). However, there is wide uncertainty around the relationship, particularly at high prevalence. Under alternative model assumptions, the predicted incidence can decrease at higher prevalences (Supplementary Fig. 1). For prevalences below 50%, the curve for all ages for daily ACD is somewhat higher than that obtained with the empirical statistical model of Patil *et al*.^10^ (Fig. 3b), which estimated incidence as detected by weekly ACD, although there is a substantial overlap in the uncertainty intervals for both models. As expected, clinical incidence is highest in the under-five age group. Supplementary Fig. 2 shows that the model-predicted incidence in under-fives is modified somewhat by seasonally varying transmission, but that the magnitude and direction of the change depend on assumptions about how immunity to disease is acquired in response to exposure, which are difficult to quantify.

### Age profile of disease

Figure 3c shows how age at which cases occur is predicted to change with declining transmission intensity. At high transmission (60% parasite prevalence in 2–10 year olds), 57% (95% CrI: 51–64) of cases are predicted to be in children under 5 years of age. At lower transmission, there is a gradual shift so that at a parasite prevalence of around 20% in 2–10 year olds, only 21% (95% CrI: 17–25) of cases are in children under 5 years of age with another 22% in children aged 5–10 years (95% CrI: 20–23). At low transmission (parasite prevalence of 5% in 2–10 year olds), 61% (95% CrI: 58–63) of cases are in children over 15 years of age, against 10% (95% CrI: 9–11) in under-fives.

### Reporting method

Figure 3d demonstrates how reported clinical incidence can vary according to case-reporting methods. Assuming that daily ACD picks up the true incidence in the population, we estimate that 72% (95% CrI: 46, 92) of cases are detected through weekly ACD and 34% (95% CrI: 12, 86) through PCD, although the estimate for weekly ACD is primarily influenced by the prior distribution (Supplementary Table 1). As our definition of PCD includes research studies, the true proportion of cases reported through the health system via PCD may be substantially lower than this estimate.

### Burden of disease

Combining our model with spatially stratified estimates of parasite prevalence from 2010, we obtain an overall estimate of 252 million (95% CrI: 171–353 million) cases of malaria in Africa in 2010 that would be detected with daily ACD or 178 million (95% CrI: 108–274 million) cases under weekly ACD (Table 2). These all-age estimates are a little lower than those reported by Hay *et al*.^8^, which are based on the empirical incidence–prevalence relationship estimated by Patil *et al*.^10^ and on prevalence estimates for 2007. Note that there is a substantial overlap between this study and ours in the data used to define the incidence–prevalence relationship, and in the parasite prevalence data. Conversely, our estimates are somewhat higher than those estimated using the WHO methodology in 2009, although in both cases there is a substantial overlap in the uncertainty intervals.

**Figure 4:**
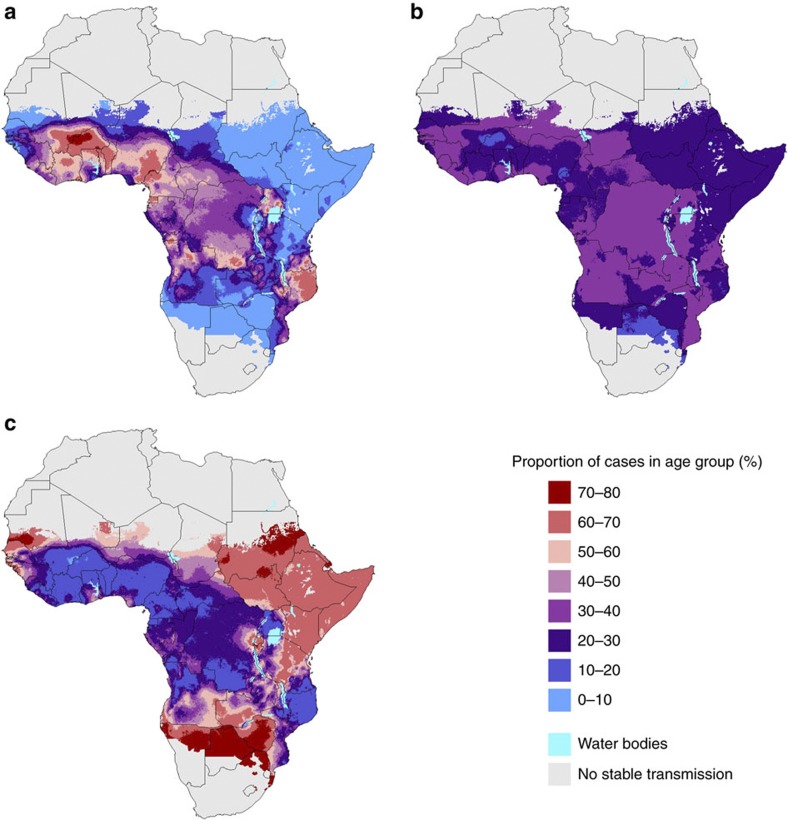
Age distribution of cases across Africa. (**a**) 0–5 years old; (**b**) 5–15 years old; (**c**) over 15 years old.

A much lower number of cases (85 million, 95% CrI: 31–213 million) are predicted to be captured under PCD, explaining part of the discrepancy between burden estimates and figures reported by countries. Overall, we estimate that 48% (95% CrI: 45–52) of cases in 2010 were in children under 5 years of age, although this average figure masks wide spatial heterogeneity (Fig. 4). Reflecting the decrease in malaria transmission that has occurred over the past decade, our estimates suggest that only in parts of west and central Africa and northern Mozambique, along with smaller pockets elsewhere, is the burden of disease now concentrated in the under-fives. In contrast, in areas in which burden has been reduced or has always been low (such as Kenya, much of Tanzania and the Horn of Africa), fewer than 20% of cases are in under-fives, and the remainder are spread across all ages. The proportion of cases in school-age children is less variable, usually between 20 and 40%, being highest in areas of medium transmission (Fig. 4b). It should be noted that uncertainty in the underlying parasite prevalence data leads to substantial uncertainty in the age distribution of cases in any given location (Supplementary Figs 3 and 4).

### Changing transmission

**Figure 5:**
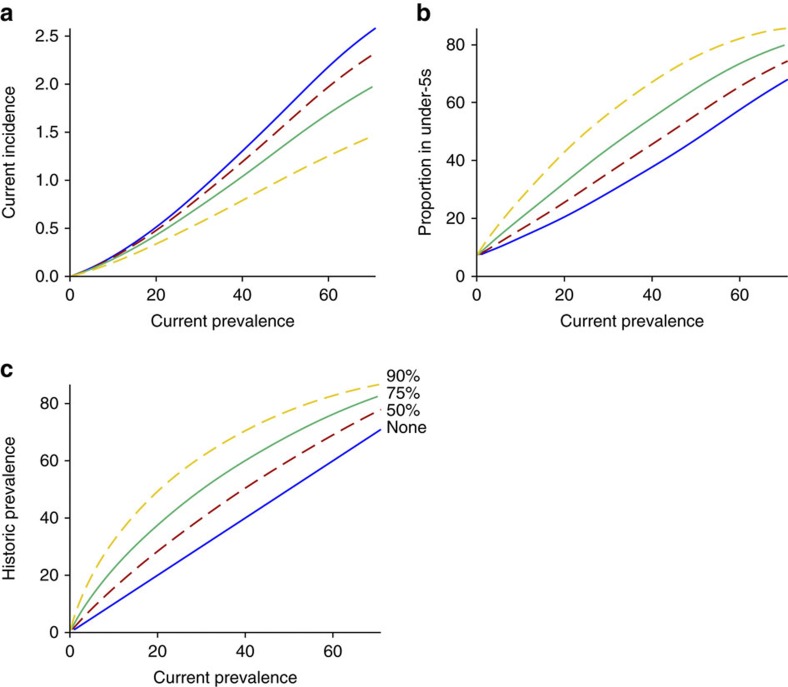
The relationship between incidence and prevalence, if transmission has recently declined. Panels show (**a**) incidence in under-fives against prevalence in 2–10 year olds, and (**b**) the proportion of cases in under-fives, following a change in transmission. (**c**) The prevalence before the decline in EIR plotted against current prevalence. Each coloured line is for a different reduction in EIR over the previous 10 years from 0 to 90%, as marked in panel **c**.

These estimates assume stable endemic transmission, whereas in fact in many areas prevalence has recently declined. Figure 5a,b shows our model-predicted relationship between incidence in under-fives, the proportion of cases that occur in under-fives and prevalence in 2–10 years olds, if transmission has recently declined. The blue line is the equilibrium solution from Fig. 3a. The other lines show a linear decrease in EIR over the previous 10 years, reducing by a maximum of 90%. Figure 5c illustrates what these reductions in EIR mean in terms of reduced prevalence. If prevalence has recently declined, then the incidence is lower for a given current prevalence because of pre-existing immunity. For the same reason, the proportion of all cases that occur in under-fives is higher, if transmission has recently declined. However, these results are dependent on the duration of naturally acquired immunity, which is not well understood.

## Discussion

By fitting a transmission model to data collected from sites covering a wide range of transmission intensities, we have obtained a biologically motivated functional relationship between prevalence of *Plasmodium falciparum* infection and incidence of uncomplicated disease. This allows for changing age patterns of disease to be characterized. Our results show a similar relationship between parasite prevalence and disease incidence to previous work, with disease incidence increasing with increasing prevalence^9^,^10^. We do not predict a decrease in disease incidence at high transmission intensity, but sparse and noisy data for high prevalence settings mean that the relationship becomes more uncertain in this regime. Hence, a plateau or slight decrease at high prevalence cannot be ruled out. Indeed, all of our estimates have a high degree of associated uncertainty, in part owing to unexplained variation between studies.

Our results illustrate that the method of case detection (active or passive) and frequency of active detection have an important impact on the incidence estimates. It has been argued that estimates of true burden should be based on ACD rather than PCD^12^. Our results suggest that PCD may only detect ~34% (95% CrI: 12, 86) of cases that would be detected through daily ACD and 49% (95% CrI: 16, 127) of cases detected through weekly ACD. Although there is a high degree of uncertainty in these sensitivity estimates, they are comparable to a published estimate of 27% obtained by comparing ACD with PCD in geographically well-defined catchment areas in Kenya^13^. Furthermore, it is estimated in the World Malaria Report 2011 that confirmed malaria cases comprise 11% of the total incidence in the African region^14^. Being able to provide estimates of disease burden based both on ACD and PCD could be beneficial in understanding the variation between burden estimates and the case numbers reported by countries. However, even the PCD estimates used here are from carefully monitored cohort studies, and so may well reflect detection of a higher proportion of cases than might be reported via existing health systems. Furthermore, there was substantial remaining variation in disease incidence between the studies that was not explained by the case detection method. We did not account for the possibility that daily ACD may itself affect transmission, as it is difficult to know what effect this could have. It may be that such close monitoring and likely high treatment rates will lead to a lower prevalence and thus higher incidence for a given prevalence. Although we incorporated the effect of routine treatment-seeking on transmission, the data on access to treatment are uncertain being based on estimates of the proportion of all fevers in under-fives treated with an antimalarial drug. Hence, uncaptured variation in treatment rates could explain some of the additional variability in the data.

An advantage of using a mechanistic model of malaria transmission is that we can estimate how the age pattern of clinical disease varies with transmission intensity. If transmission is stable, cases are concentrated in the first few years of life at high endemicity, but in older ages at lower endemicity. Our model captures this peak shift, enabling an estimate to be made of the incidence in each age group for any level of endemic transmission. However, in some settings, we were unable to capture the magnitude or age of the observed peak. This may be due to variation in reporting between studies, including variation by age, or to seasonality in exposure that was not included in the model fitting for computational ease, but which may modify the rate of acquisition of immunity. An additional limitation is that only 14 studies were analysed, with varying study designs, including different definitions for an episode of malaria. Estimates of disease burden could be improved by fitting to larger data sets from a range of transmission settings. In particular, a standardised design and definition could substantially reduce the residual uncertainty in the relationship between transmission intensity and disease incidence. One possible source of such data is from the control arm of trials that are increasingly undertaken in multiple sites. We did not account for dynamically changing conditions, but instead treated the current prevalence as if it were the endemic prevalence, both in the model-fitting estimates and in the burden estimates. As our fitted transmission model incorporates the acquisition and loss of naturally acquired immunity, and can be linked to a wider existing suite of intervention models^11^, it can be used to estimate how the burden and age pattern of disease incidence may change in the future as scale-up of interventions is sustained and enhanced. Our results demonstrate that if transmission has recently declined rapidly and naturally acquired immunity is long-lived, the majority of cases may still be in the young, and the model-predicted incidence will be lower for a given prevalence than if transmission is in equilibrium. As immunity is gradually lost, we would then predict a shift in these cases to older ages such that eventually the age distribution of cases would match those presented in the main text. Predicting when this shift would occur is difficult as the time-scale and magnitude of any rebound in incidence as immunity wanes following a reduction in transmission are poorly understood, and thus estimates of impact must be interpreted with this uncertainty in mind.

Capturing the changing age pattern of disease as transmission declines is important in estimating the burden of clinical disease. Of the principal methods currently used to calculate burden, only that used by the WHO takes into account age, although it does not incorporate dynamic shifts in the age distribution of disease resulting from declining transmission. Age-based estimates of death rates attributable to malaria have also been published based on verbal autopsy data, with a controversially large estimate for the burden in adults that does not appear to be borne out in case reports^15^,^16^,^17^. One possible reason for this may be that the method used in the Global Burden of Disease study does not account for changing immunity in the population, and hence deaths in adults could be predicted in large areas of sub-Saharan Africa in which transmission has recently declined rapidly but in which, in reality, the adult population still remains highly immune. However, deaths from malaria typically occur at a younger age than uncomplicated cases, and hence are not directly comparable to our estimates of clinical disease burden.

The age distribution of clinical cases is also important when targeting interventions. For example, seasonal malaria chemoprevention is currently recommended for children aged from 3 months to 5 years based on trial data from areas with high seasonal endemic transmission^18^, but it may also be beneficial for older children in areas where transmission has been reduced, as has recently been shown in Senegal^19^. Other interventions, including access to free treatment, long-lasting insecticide-treated net distribution and the new RTS,S vaccine, are frequently or will be targeted at children under 5 years of age^20^,^21^,^22^, although universal coverage of LLINs is now promoted.

Our results suggest that in much of Africa, there has been or will be a shift in cases to older children and adults, but that there is wide between- and within-country variability in the age groups at the highest risk. Hence, age-targeted policies may increasingly need to be refined between and even within countries. To underpin such decisions, and thereby make the best use of the limited resources available, country control programmes need estimates of the age distribution of cases. In some countries case reporting will be sufficient. However, because the population accessing the public sector is heterogeneous and often only represents a minority of total cases^5^, and treatment-seeking behaviour may vary with age^23^, in other countries case reports may not give a reliable picture of the true age-burden of disease. The methodology and estimates presented here therefore add to the information that can be used at country level to aid such decision-making.

## Methods

### Clinical incidence data

We extracted data on the incidence of clinical disease due to *P. falciparum* and, where recorded, parasite prevalence, stratified by age in sub-Saharan Africa in the period 1990–2005 from two recent review papers^2^,^10^. We restricted our analysis to studies that included data in children and where the numbers of person-years at risk and cases in each age group were reported or could be calculated. The majority of studies reported incidence data over a whole number of years. Where they did not we adjusted the time at risk using the time of the malaria season as reported in each paper. For example, if a study lasted for 6 months and covered an entire transmission season, we multiplied the reported time at risk by two, assuming that for the other 6 months the incidence would be low.

A variety of definitions of clinical malaria were reported, falling into two groups: malaria symptoms (fever and sometimes other symptoms) plus any parasitaemia^24^,^25^,^26^,^27^,^28^,^29^,^30^,^31^; or malaria symptoms plus parasite density above a certain threshold. The threshold was either fixed^32^; dependent on transmission intensity, increasing at higher transmission^33^; or dependent on age, with the thresholds decreasing with age, except possibly in the first year of life^34^,^35^,^36^,^37^. In one study that had both a fixed and an age-dependent threshold, we used the former^38^.

Those studies using ACD were categorized into daily or weekly ACD. Studies with less frequent active detection were excluded, hence in the remainder of the studies cases were recorded on presentation at the health facility (PCD). We took the true incidence to be that given by daily ACD. The data sources for clinical incidence are summarized in Table 1. Additional data on parasite prevalence stratified by age were incorporated in the model fitting. This included those studies previously used in our transmission model fitting^11^, the Garki data stratified by clusters of villages^39^ and prevalence data reported at the same site and time as the clinical disease data (Table 1). We ignored seasonal variation in parasite prevalence, but excluded studies where it was clear that data were only collected in the high season.

### Transmission model

**Figure 6:**
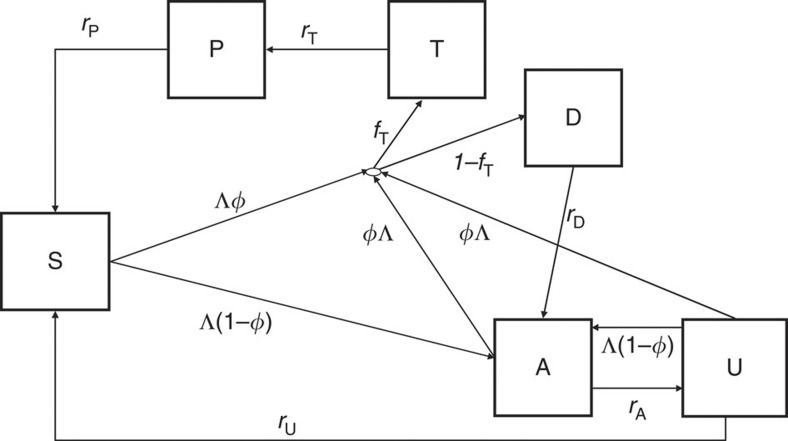
Model flow diagram. S, susceptible, D, clinical disease, A, asymptomatic patent infection, U, subpatent infection, T, treated and P, prophylaxis. The parameters are defined in the text.

A flow diagram of the model is shown in Fig. 6, and further details are given in the Supplementary Methods. In brief, susceptible individuals (state S) become infected at a rate Λ determined by the EIR, and following a latent period either develop disease with probability *φ* or else develop asymptomatic patent infection (state A), where the probability *φ* is modified by clinical immunity. Those that develop disease can become treated (state T) and enter a protected state P to reflect the protective effect of the first-line therapy, or enter state D and then recover naturally to progress to asymptomatic infection. Asymptomatic individuals move from patent infection A to subpatent infection U before recovering to the susceptible state. Superinfection is possible from the asymptomatic states. All states are stratified by age and level of exposure to mosquitoes.

We have refined the representation of immunity in the model compared with the version published previously^11^. First, we modified the effect of antiparasite immunity. Previously its action had been modelled as reducing the duration of patent infection, but we instead model it as modifying the slide detectability of patent infection, as suggested by recent analyses of genotyping data^40^. Second, we constrained the effect of anti-infection immunity to give a reduction in infection risk of no more than 50% of that experienced by a non-immune person, based on RTS,S vaccine trials^22^. Finally, to allow a more flexible relationship between EIR and clinical incidence, the probability of clinical disease was allowed to be less than one in the absence of immunity, whereas we previously assumed that all new infections cause acute disease in people without prior exposure to malaria. These changes improve the fit to the clinical incidence data, but do not substantially change the dynamics of the transmission model compared with the previously published version.

### Model fitting

The endemic equilibrium of the non-seasonal model was fitted using Bayesian methods to the prevalence and incidence data. To incorporate the variation seen in the incidence of disease between different studies in areas with similar EIR, we included study-level random effects for the clinical incidence and prevalence data. In the model, only one episode of clinical disease can occur with each new infection. This is then fitted to the clinical incidence data as measured using each study’s definition of an episode of clinical malaria. Data on human infectiousness to mosquitoes according to age were also included in the model fitting, plotted in Supplementary Fig. 5.

For each study site, we sought an estimate of the EIR, from the same study year and location if available, otherwise from the same region of the country. We accounted for uncertainty in EIR estimates by assuming the prior uncertainty in EIR increased with the spatial or temporal distance between the locations at which the EIR was measured and where the corresponding incidence or prevalence data were collected. Supplementary Table 2 lists the prior s.d.s chosen. We also used informative prior distributions for the treatment rates for each study (Table 1). Data on treatment rates by country were obtained from the relevant Demographic Health Surveys and Malaria Indicator Surveys^41^. Prior distributions for the other parameters in the model are similar to those previously reported^11^. Full details of all prior distributions, model likelihoods and parameter estimates are given in the Supplementary Methods and Supplementary Table 1. Estimates of all quantities are posterior medians, with 95% credible intervals.

### Burden estimation

We combined our estimate of the relationship between parasite prevalence and the incidence of clinical disease with published spatially stratified estimates of the parasite prevalence in 2–10 year olds in 2010 (ref. 42) to obtain spatially stratified estimates of the incidence of disease by age and case detection methods. In each 5 km^2^, we calculated the clinical incidence rate in 5-year age groups, taking account of the uncertainty both in our model estimates and in the parasite prevalence in that square. We then multiplied the incidence rate by an estimate of the population in 2010 in each age group to obtain an estimate of the overall incidence in each age group. The 2010 population was derived from three sources: landscan estimates of the population at 5 km resolution in 2007 (ref. 43); country-specific estimates of the age distribution from the UN; and population growth rates from the World Bank. The end result is a posterior distribution of the number of cases and the proportion in each age group, both at 5 km resolution and aggregated across Africa. The aggregate estimates do not account for correlation in the estimates of parasite prevalence between neighbouring locations, hence the uncertainty is likely to be underestimated, as shown in (ref. 44). For model predictions and burden estimation we assumed a treatment rate of 40%, informed by the Demographic Health Surveys data. Results with different treatment rates are given in Supplementary Table 3 and Supplementary Fig. 6.

## Additional information

**How to cite this article:** Griffin, J. T. *et al*. Estimates of the changing age-burden of *Plasmodium falciparum* malaria disease in sub-Saharan Africa. *Nat. Commun.* 5:3136 doi: 10.1038/ncomms4136 (2014).

## Supplementary Material

Supplementary Information Supplementary Figures 1-6, Supplementary Tables 1-3, Supplementary Methods and Supplementary References

## Figures and Tables

**Table 1:**
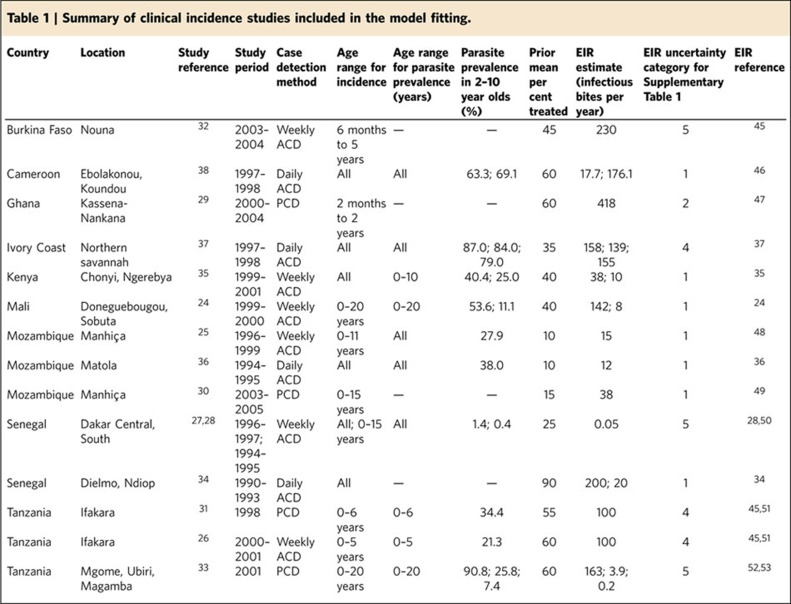
Summary of clinical incidence studies included in the model fitting.

**Table 2:** Estimates of the annual number of cases of clinical malaria due to *P. falciparum* in Africa.

**Source**	**Cases of clinical malaria (millions)**	
	**Estimate**	**95% Uncertainty interval**
*Our estimates for 2010*		
With daily ACD	252	171, 353
With weekly ACD	178	108, 274
With PCD	85	31, 213
*Cibulskis et al*.^7^		
Estimate for 2009*	173	107, 243
*Hay et al*.^8^		
Estimate for 2007†	271	241, 301

^*^Estimates for sub-Saharan Africa excluding Sudan, south Sudan, Somalia and Djibouti.

^†^Estimate for Africa+region, covering Africa and the Arabian Peninsula.
