# Supplementary material for: Estimates of the changing age-burden of Plasmodium falciparum malaria disease in sub-Saharan Africa
Source: Nat Commun. 2014 Feb 11;5:3136. doi: 10.1038/ncomms4136 (PMC3923296; doi:10.1038/ncomms4136)
Supplement: Supplementary Information — Supplementary Figures 1-6, Supplementary Tables 1-3, Supplementary Methods and Supplementary References [file ncomms4136-s1.pdf]

Supplementary Figures

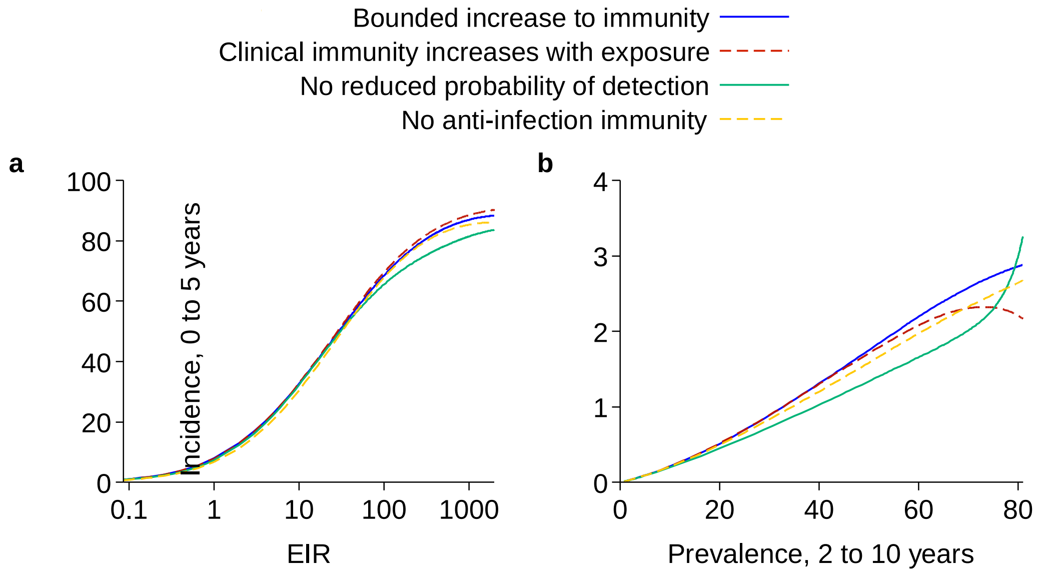

Supplementary Figure 1. The impact of different choices of immunity model on the relationship between prevalence, incidence and EIR.

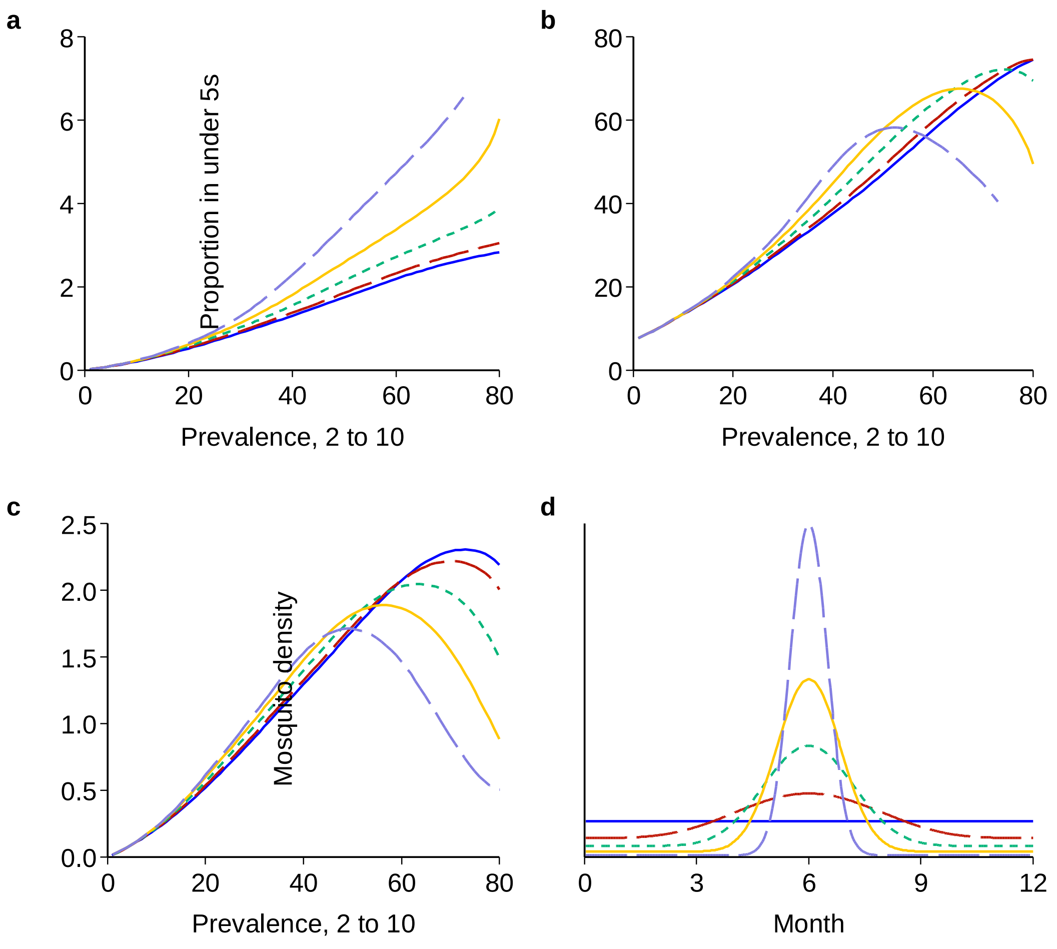

Supplementary Figure 2. Incidence-prevalence relationship (a) and proportion of cases in under-fives (b) with varying degrees of seasonality; incidence-prevalence relationship if immunity to clinical disease increases with every exposure (c); and seasonal curves of mosquito numbers (d). The colours of the lines in parts a-c correspond to the curves in part d.

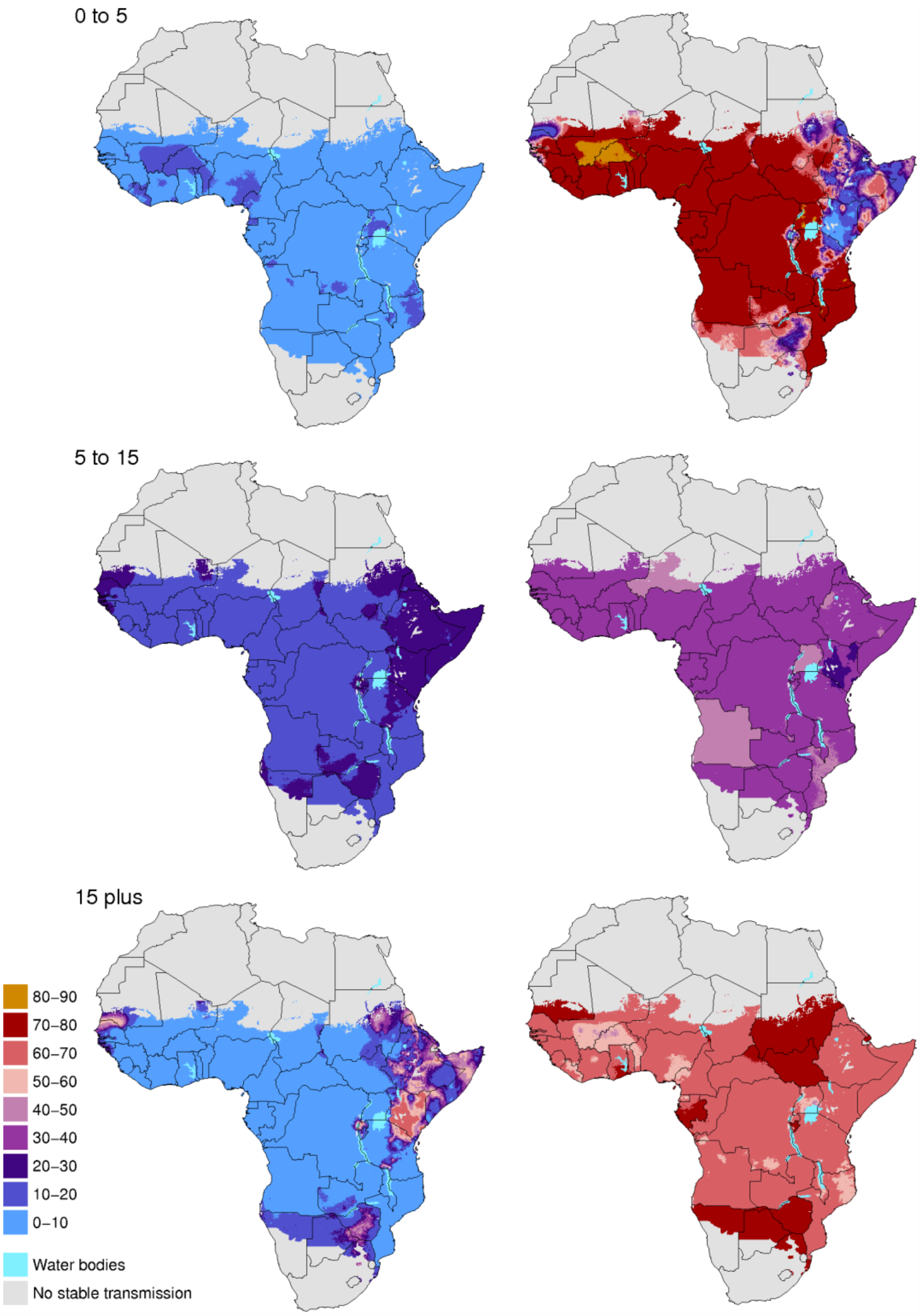

Supplementary Figure 3. Lower and upper 95% credible intervals (left and right respectively) for the proportion (%) of clinical cases in each age group, using the Malaria Atlas Project posterior distribution of prevalence.

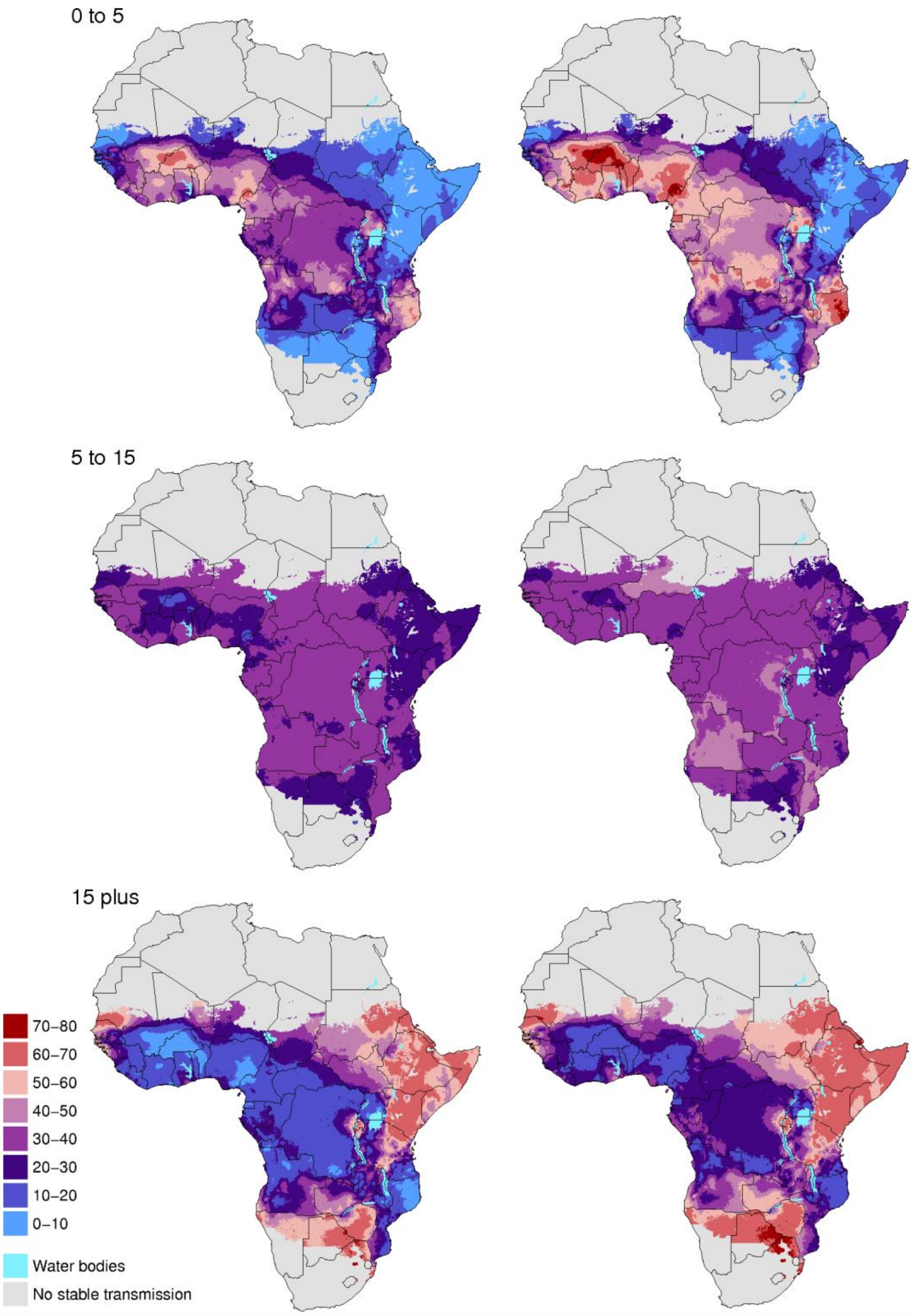

Supplementary Figure 4. Lower and upper 95% credible intervals (left and right respectively) for the proportion (%) of clinical cases in each age group, using the posterior mean Malaria Atlas Project prevalence.

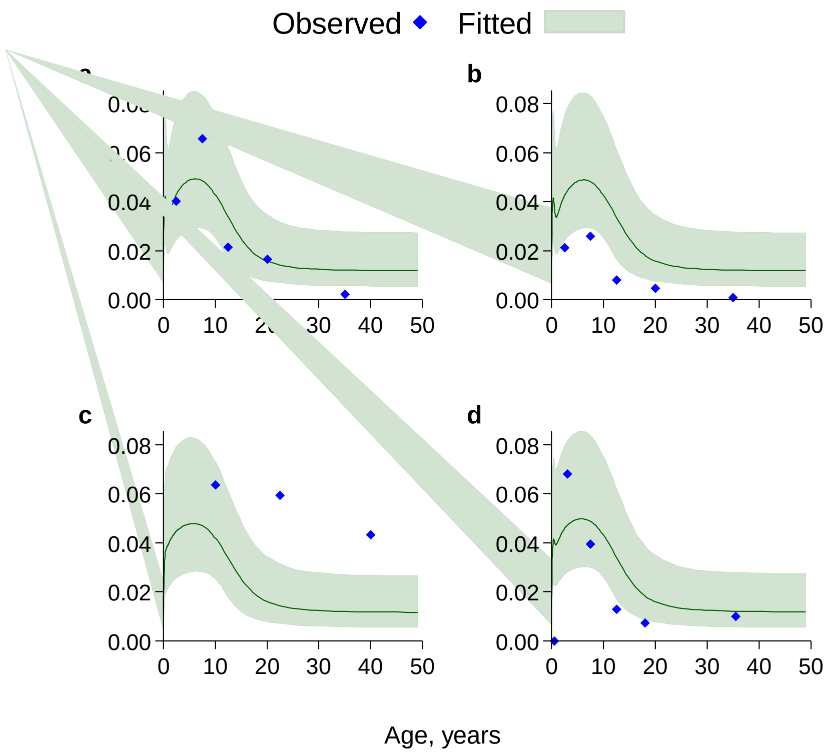

Supplementary Figure 5. Observed and fitted proportions of mosquitoes infected, with references for each study: (a) Bondi village[54]; (b) Menang village[54]; (c)[55]; (d)[56]

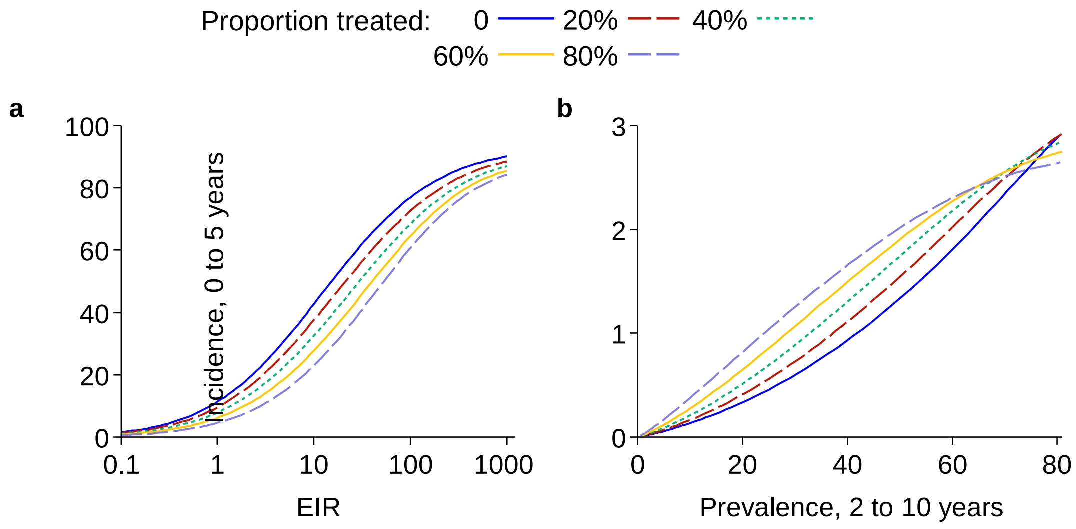

Supplementary Figure 6. Prevalence, incidence and EIR according to proportion treated.

Supplementary Tables

Supplementary Table 1. Model parameter estimates and prior distributions.

Parameter description	Symbol	Prior median and 95% interval	Posterior median and 95% credible interval; or fixed value	References
**Age and heterogeneity**				
Age-dependent biting parameter	ρ	Fixed	0.85	[57,58]
Age-dependent biting parameter	a_0	Fixed	8 years	
Variance of log of heterogeneity in biting rates	σ^2	Fixed	1.67	[59]
**Human infection durations (all in days)**				
Latent period	d_E	Fixed	12	[60]
Patent infection	d_I	Fixed	200	[60,61]
Clinical disease (treated)	d_T	Fixed	5	[62]
Clinical disease (untreated)	d_D	Fixed	5	[63]
Sub-patent infection	d_U	67 (30, 100)	110 (87, 131)	
Prophylaxis following treatment	d_P	Fixed	25	[64]
**Infectiousness to mosquitoes**				
With no immunity, or with untreated disease	c_D	0.24 (0.06, 0.52)	0.068 (0.039, 0.122)	[65]
After treatment	c_T	Fixed	$0.32 \times c_D$	[66]
In sub-patent infection	c_U	0.004 (0.0004, 0.016)	0.0062 (0.00056, 0.018)	
Relates infectiousness to probability of detection	γ_I	3.6 (0.16, 15)	1.82 (0.603, 8.54)	
**Immunity reducing probability of detection**				
Probability with maximum immunity	d_1	0.25 (0.13, 0.39)	0.161 (0.089, 0.240)	[40]
Inverse of decay rate	d_{ID}	Fixed	10 years	
Scale parameter	I_{D0}	21 (1.3, 89)	1.58 (0.22, 6.18)	
Shape parameter	κ_D	2.01 (0.88, 3.06)	0.477 (0.280, 0.826)	
Duration in which immunity is not boosted	u_D	5.85 (0.85, 19.5)	9.45 (3.57, 19.2) days	

Scale parameter relating age to immunity	a_D	9.9 (3.3, 17.3)	21.9 (19.2, 25.3) years	
Parameter relating age to immunity	f_{D0}	0.5 (0.025, 0.975)	0.0071 (0.00035, 0.0259)	
Shape parameter relating age to immunity	γ_D	1.9 (0.36, 4.3)	4.81 (3.79, 6.26)	
PCR prevalence parameters	α_A	0.5 (0.025, 0.975)	0.757 (0.545, 0.940)	
	α_U	0.84 (0.12, 2.8)	0.186 (0.026, 0.668)	
**Immunity reducing probability of infection**				
Probability with no immunity	b_0	0.5 (0.05, 0.95)	0.590 (0.389, 0.845)	
Maximum relative reduction	b_1	Fixed	0.5	22
Inverse of decay rate	d_B	Fixed	10 years	
Scale parameter	I_{B0}	43 (3.5, 132)	43.9 (20.1, 120)	
Shape parameter	κ_B	2.01 (0.88, 3.06)	2.16 (1.22, 2.93)	
Duration in which immunity is not boosted	u_B	5.85 (0.85, 19.5)	7.20 (2.63, 15.0) days	
**Immunity reducing probability of clinical disease**				
Probability with no immunity	ϕ_0	0.82 (0.52, 0.97)	0.792 (0.548, 0.961)	67
Maximum relative reduction	ϕ_1	0.39 (0.02, 0.85)	0.00074 (0.00005, 0.0025)	
Inverse of decay rate	d_C	Fixed	30 years	68
Scale parameter	I_{C0}	95 (5.8, 316)	18.0 (11.9, 26.7)	
Shape parameter	κ_C	2.01 (0.88, 3.06)	2.37 (1.99, 2.86)	
Duration in which immunity is not boosted	u_C	5.85 (0.85, 19.5)	6.06 (2.82, 11.1) days	
New-born immunity relative to mother's	P_M	0.5 (0.025, 0.975)	0.774 (0.536, 0.981)	
Inverse of decay rate of maternal immunity	d_M	205 (102, 362)	67.7 (59.0, 79.4) days	69
**Case detection: recorded incidence relative to daily ACD**				
Weekly ACD	r_W	Beta: 0.76 (0.48, 0.94)	0.723 (0.460, 0.923)	70
PCD	r_P	0.5 (0.025, 0.975)	0.342 (0.120, 0.860)	

Supplementary Table 2. Assumed prior uncertainty for EIR estimates.

Relationship between clinical incidence data and estimate of EIR	*EIR uncertainty category for Table 1*	*Standard deviation for log-normal prior distribution*
Same time and place, i.e. the same village, town or district of a city	1	0.3
Same time but different place	2	0.4
Same place but different year	3	0.5
Different place and different year	4	0.6
Different place and different year, with substantial disagreement between estimates	5	0.8

Supplementary Table 3. Estimated Africa-wide burden and age-distribution of clinical malaria with daily ACD according to proportion treated.

Proportion treated (%)	Number of cases (millions)		Percent of cases in under-fives	
	Estimate	95% CrI	Estimate	95% CrI
0	236	163, 324	45.5	41.5, 49.7
20	244	167, 338	47.3	44.0, 51.1
40	252	171, 353	48.4	45.2, 51.9
60	261	176, 372	48.9	45.5, 52.5
80	272	180, 395	49.1	45.1, 52.7

Supplementary Methods

Transmission model

The transmission model which we fitted to the prevalence and clinical incidence data was in most respects the same as a previously published model[11], but is described here for completeness. At each point in time people can be in one of six states: susceptible (S), treated clinical disease (T), untreated clinical disease (D), asymptomatic infection which may be detected by microscopy (A), sub-patent infection (U) and protected by a period of prophylaxis from prior treatment (P). Latent liver stage infection is modelled as a delay in the force of infection of duration d_E. People move between these states as shown in Figure 6. The partial differential equations for the human dynamics are as follows, with t representing time and a age,

$$
\begin{aligned}
\frac{\partial S}{\partial t}+\frac{\partial S}{\partial a} &= -\Lambda S + P/d_P + U/d_U \\
\frac{\partial T}{\partial t}+\frac{\partial T}{\partial a} &= \phi f_T \Lambda (S+A+U) - T/d_T \\
\frac{\partial D}{\partial t}+\frac{\partial D}{\partial a} &= \phi(1-f_T)\Lambda(S+A+U) - D/d_D \\
\frac{\partial A}{\partial t}+\frac{\partial A}{\partial a} &= (1-\phi)\Lambda(S+U) + D/d_D - \phi\Lambda A - A/d_A \\
\frac{\partial U}{\partial t}+\frac{\partial U}{\partial a} &= A/d_A - U/d_U - \Lambda U \\
\frac{\partial P}{\partial t}+\frac{\partial P}{\partial a} &= T/d_T - P/d_P
\end{aligned}
\tag{1}
$$

Λ is the force of infection, ϕ is the probability of clinical disease upon infection, f_T is the probability that clinical malaria is effectively treated, and each d is the mean duration in a state . Rather than numerically solve the partial differential equations, we stratified the infection states by age, and also by degree of exposure to mosquitoes. The equilibrium states for a given EIR can then be found by iteratively going through the age groups from youngest to oldest.

Heterogeneity in biting rates

We assume that each person has a relative biting rate ζ following a log-normal distribution between people with parameters $-\sigma^2/2$ and σ, so that ζ has a mean of 1. With a mean EIR for adults of ε_0, the EIR ε and force of infection Λ at age a are given by

$$\varepsilon = \varepsilon_0 \zeta \left(1 - \rho \exp(-a / a_0)\right)$$
$$\Lambda = \varepsilon b \qquad (2)$$

where b is the probability of infection if bitten by an infectious mosquito, and ρ and a_0 determine how the biting rate changes with age. To model this heterogeneity using a compartmental model, the infection states are stratified by exposure as well as age. Suppose that there are n exposure categories. Let $x_1,...,x_n$ and $w_1,...,w_n$ be the Gauss-Hermite abscissas and weights for integrating a function multiplied by a standard normal probability density[71]. We take a proportion w_i of the population to be in exposure category i and set the relative biting rate in this category to be

$$\zeta_i = \exp\left(-\sigma^2 / 2 + \sigma x_i\right) \qquad (3)$$

Then the EIR in the category i is

$$\varepsilon_i = \varepsilon_0 \zeta_i \left(1 - \rho \exp(-a / a_0)\right) \qquad (4)$$

Immunity functions

We consider three points at which immunity may act:

- a reduction in the probability of infection following an infectious challenge (pre-erythrocytic immunity, I_B);
- a reduction in the probability of clinical disease upon infection (acquired and maternal clinical immunity, I_{CA} and I_{CM} respectively);
- and blood stage immunity that both reduces the probability of detection and reduces infectiousness to mosquitoes (detection immunity, I_D).

In our previously published model, the effect of blood-stage immunity was instead to reduce the duration of infection.

The three types of acquired immunity increase with exposure or wane as follows, starting from zero at birth:

$$\frac{\partial I_B}{\partial t} + \frac{\partial I_B}{\partial a} = \frac{\varepsilon}{\varepsilon u_B + 1} - I_B / d_B$$
$$\frac{\partial I_{CA}}{\partial t} + \frac{\partial I_{CA}}{\partial a} = \frac{\Lambda}{\Lambda u_C + 1} - I_{CA} / d_C \qquad (5)$$
$$\frac{\partial I_D}{\partial t} + \frac{\partial I_D}{\partial a} = \frac{\Lambda}{\Lambda u_D + 1} - I_D / d_{ID}$$

Here, each u represents the time during which immunity cannot be boosted after a previous boost and each d governs the duration of immunity. The immunity functions are stratified by age and exposure to mosquitoes.

The probabilities of infection, detection and clinical disease are given by Hill functions, as these have a sufficiently flexible shape for a quantity which is assumed to decrease from its value with no immunity to a minimum value with high immunity.

The probability of infection is:

$$b = b_0 \left(b_1 + \frac{1 - b_1}{1 + \left(I_B / I_{B0} \right)^{\kappa_B}} \right) \tag{6}$$

b_0 is the probability with no immunity, $b_0 b_1$ is the minimum probability, and I_{B0} and κ_B are scale and shape parameters.

Maternal clinical immunity I_{CM} is assumed to be at birth a proportion P_M of the acquired immunity of a 20 year-old and to decay at rate $1/d_M$. The probability of acquiring clinical disease upon infection is then

$$\phi = \phi_0 \left(\phi_1 + \frac{1 - \phi_1}{1 + \left((I_{CA} + I_{CM}) / I_{C0} \right)^{\kappa_C}} \right) \tag{7}$$

where ϕ_0 is the probability with no immunity, $\phi_0 \phi_1$ is the minimum probability, and I_{C0} and κ_C are scale and shape parameters. Previously we took ϕ_0 to be 1, i.e. all new infections cause disease in the absence of immunity, and ϕ_1 to be 0, but here we fit both parameters.

The probability that an asymptomatic infection (state A) will be detected by microscopy is

$$q = d_1 + \frac{(1 - d_1)}{\left(1 + (I_D / I_{D0})^{\kappa_D} f_D \right)} \tag{8}$$

d_1 is the minimum probability, and I_{D0} and κ_D are scale and shape parameters. f_D is a purely age-dependent function given by $f_D = 1 - (1 - f_{D0}) / \left(1 + (a / a_D)^{\gamma_D} \right)$ at age a, with parameters f_{D0}, a_D and γ_D. Two additional parameters, α_A and α_U,

determine the probabilities that states A and U are detected by PCR, which are given by q^{α_A} and q^{α_U} respectively.

Infectiousness to mosquitoes

The lower probability of detection is assumed to be due to a lower parasite density, which also reduces onward infectivity. In state D (clinical disease) and state U (sub-patent infection), infectiousness is c_D and c_U respectively. In state A, infectiousness is given by $c_U + (c_D - c_U)q^{\gamma_I}$ where q is the probability of detection of parasites and γ_I is a parameter to be estimated. Following treatment, infectivity is c_T.

Model likelihood

The model was fitted to data in a Bayesian framework using Markov Chain Monte Carlo (MCMC). Suppose that within a study, there are sites indexed $j = 1,...,m$. Let $k = 1,...,4$ index the age groups 0 to 2 years, 2 to 5, 5 to 15 and 15 plus. Within these groups, suppose that the study presented data in age groups $i = 1,...,s_k$. We assume that there is a study-level random effect u and random effects v_{jk}, with $u \sim Normal\left(0, \sigma_C^2\right)$ and $v_{jk} \sim Gamma(1/\alpha_C, \alpha_C)$.

For a given set of parameters, including the EIR for each site, let the model-predicted incidence of clinical malaria in site j and age group i be μ_{ji}, and the person-time at risk and number of events be T_{ji} and y_{ji} respectively. We also include a parameter r for the method of case detection, with $r = 1$ for daily ACD and less than 1 for weekly ACD or PCD. We then assume that

$$y_{ji} \sim Poisson(re^u v_{jk(i)} T_{ji} \mu_{ji}) \qquad (9)$$

The rationale for this formulation of the age-specific random effects is that if the age groups $k = 1,...,4$ coincided with the age groups in the study, then the Poisson-Gamma model would be equivalent to a negative binomial model (conditional upon the random effects u). However, there was finer age stratification in some studies than others, and simply having a negative binomial likelihood for each age group i would give different statistical weight to a study depending on how finely the data were grouped.

The overall likelihood for clinical incidence data in a study is thus

$$L = f\left(u \mid -\sigma_C^2/2, \sigma_C^2\right) \prod_{j=1}^{m} \prod_{k=1}^{4} g(v_{jk} \mid 1/\alpha_C, \alpha_C) \prod_{i=1}^{s_k} P(y_{ji} \mid ruv_{jk} T_{ji} \mu_{ji}) \qquad (10)$$

where f is a log-normal probability density, g is a Gamma probability density and P is a Poisson probability. The random effects v_{jk} can be analytically integrated out of this likelihood[72], and we numerically integrated the random effects u out by adaptive Gauss-Hermite quadrature. Using numerical integration rather than sampling u in the MCMC fitting has a number of advantages: it reduces the number of quantities sampled; the EIRs for each site were sampled, and these are correlated with the random effects; and the model equilibrium only needs to be found once, and then in the numerical integration the same equilibrium value is used repeatedly. The accuracy of the integration was checked by increasing the number of quadrature points and ensuring that the change in the posterior parameter estimates was negligible.

Let the model-predicted prevalence of infection in site j and age group i be p_{ji}, and the number sampled and positive be n_{ji} and x_{ji} respectively. Then we assume that there is a study-level random variable $w \sim Normal(0, {\sigma_P}^2)$, and that

$$x_{ji} \sim Beta\text{-}binomial\left(q_{ji}\theta, (1-q_{ji})\theta, n_{ji}\right) \qquad (11)$$

where $q_{ji} = invlogit\left(logit(p_{ji}) + w\right)$, with $logit(p) = log\left(p/(1-p)\right)$ and $invlogit$ its inverse function, and θ is an over-dispersion parameter. We integrated the random effects w out of the likelihood by adaptive Gauss-Hermite quadrature. Data were stratified into age groups with boundaries 0, 2, 5, 10, 15, 20, 30, 40 years if they were presented in finer age groups than this: unlike clinical incidence, parasite prevalence does not have a sharp peak at young ages, hence less information is lost by grouping the data.

The infectivity parameters were fitted alongside the rest of the model. The data for infectivity consisted of three published studies in which mosquitoes were fed on a sample from the population of endemic areas, regardless of the volunteers' infection status[54-56]. In each study, numbers of mosquitoes feeding and infected were recorded by age group, and there was information on parasite prevalence. A Beta-binomial likelihood was used with over-dispersion parameter α_I.

Prior distributions and posterior estimates

The prior distribution for the log of the EIR in each of the datasets was taken as normally distributed with the standard deviation chosen according to whether or not the estimate was in the same year, and whether it was from the same village or town or from elsewhere in the same region (Supplementary Table S2).

Data on treatment rates by country were obtained from the relevant Demographic Health Surveys (DHS) and Malaria Indicator Surveys (MIS)[41]. These surveys have data on the proportion of fever cases in under-fives which were treated with an anti-malarial drug, which we took to be the treatment rate. As estimates were variable between surveys in the same country, we chose compromise values based on surveys near in time (Table 1). The prior estimate for the treatment rate was taken as 90% in the closely monitored cohorts in Dielmo and Ndiop, Senegal, and 5% for data from the Garki study. The prior distribution for the proportion of clinical cases effectively treated was a Beta distribution with mean at 75% of the DHS-derived value, and sum of Beta shape parameters = 25, where the factor 75% accounts for imperfect efficacy of pre-ACT treatments [73].

Prior distributions for all other parameters in the model are similar to those previously reported[11]. Supplementary Table S1 shows the prior and posterior estimates for all the model parameters if the parameter was estimated. Some parameters were fixed based on values from the literature, and some shape parameters were given moderately informative prior distributions in order to ensure identifiability. In particular, the parameter governing heterogeneity in biting (σ) was fixed. This parameter has a substantial impact on the basic reproduction number and hence on the effectiveness of interventions. When we tried fitting the parameter, its posterior value differed greatly from the prior value, even though it is clear that the data does not contain genuine information about heterogeneity in biting. The observed and model-predicted probability of mosquito infection by age of the human population are shown for each study in Supplementary Figure S5.

Spatial Data Sources and Modelled Relationships

The methods for estimating the Africa-wide burden of clinical malaria in 2010 based on the parasite prevalence in 2 to 10 year-olds are summarised in the main text. Here we give further details.

To estimate the population by age across sub-Saharan Africa, we started with Landscan estimates of the population at 5km resolution in 2007[43]; we then multiplied these by country-specific growth rates from the World Bank to obtain estimates of the population in 2010; and finally, used the estimates of the age distribution in each country from the UN to estimate the proportion of people in each 5km square and each five-year age group.

In each 5km square in sub-Saharan Africa we drew a sample of 10,000 prevalence values from the posterior distribution in that square: this distribution is from the model fitted by the Malaria Atlas Project (MAP)[42]. We then took a sample of 10,000 parameter sets from the posterior distribution of our fitted model, and for each pair

of prevalence value and model parameter set we calculated the incidence that would be detected by different case detection methods, by five-year age groups, using the equilibrium model solutions. We then multiplied the incidence by the estimated number of people in each age group in that 5km square, to give a posterior distribution of the number of cases in each age group. This gives a posterior distribution of the proportion of cases by age at 5km resolution, or the numbers of cases by age group can be aggregated to give a posterior distribution of the Africa-wide burden and age-distribution of cases. Our model is not well-validated for prevalences above 80%, and needs implausibly high EIRs to reach prevalences above 85%. Hence when sampling from the posterior distribution of prevalence in each square, when the sampled value was above 80%, we set the clinical incidence to be the same as it would be at a prevalence of 80%.

There is wide uncertainty in the proportion of clinical cases in each age group across Africa (Supplementary Figure S3), due primarily to the uncertainty in the underlying prevalence estimates. If instead of the full posterior distribution, we use the posterior mean prevalence from the MAP model, so that the only uncertainty is in our model estimates, the proportion of cases in each age group is much more precisely estimated (Supplementary Figure S4).

Sensitivity analysis to immunity model

The relationship between clinical incidence and prevalence is somewhat sensitive to the choice of immunity model (Supplementary Figure S1B). The model presented in the main text (blue line) assumes that immunity against clinical disease increases with exposure, but with a delay after each boost to immunity during which immunity cannot be further boosted. This delay was included for biological plausibility. In this model clinical incidence always increases with prevalence. We fitted an alternative model in which immunity to disease is boosted at every exposure, so that in equation **Error! Reference source not found.** I_{CA} increases as follows:

$$\frac{\partial I_{CA}}{\partial t}+\frac{\partial I_{CA}}{\partial a}=\Lambda-I_{CA}/d_C \qquad (12)$$

Now the fitted incidence decreases at higher prevalences (red line). The model the main text incorporates a pre-erythrocytic/liver-stage immunity against infection and also models the effect of blood-stage immunity in reducing the probability of detection, with the choice of model based on the demonstrated partial efficacy of the RTS,S vaccine and on estimates of the detectability of parasite clones from genotyping studies[22,40]. Refitting with either of these removed also changes the incidence-prevalence curves (green and yellow lines). Incorporating immunity that

reduces the duration of infection to a minimum duration of patent infection of 50 days made little difference to these relationships (not shown).

Sensitivity analysis to treatment rate

For model predictions and burden estimates, we assumed a treatment rate of 40% (the proportion of clinical cases treated with an effective drug), as the DHS and MIS surveys do not have data for every country, whereas we used country-specific data when fitting the model. A higher EIR is required to explain a given prevalence if there is a high treatment rate (Supplementary Figure S6a). Hence if the model is calibrated to match a given prevalence, there is a higher incidence if the treatment rate is higher (Supplementary Figure S6b). This results in the estimated Africa-wide burden increasing with the assumed treatment rate (Supplementary Table S3). However the estimated proportion of cases that occurs in under-fives is less sensitive to this assumption.

Sensitivity analysis to seasonal variation

Supplementary Figure S2 shows how the results are affected if there is seasonal variation in transmission, with the mosquito density varying over the year in proportion to the curves in Supplementary Figure S2d, with the same pattern repeating each year. The incidence and prevalence shown are the mean over the year. With the model presented in the main text, the incidence in under-fives is higher when there is marked seasonality than in a perennial transmission setting (Supplementary Figure S2a), whereas the proportion of cases in under-fives is less affected (Supplementary Figure S2b). In a model in which immunity to clinical disease increases in proportion to exposure (the model plotted with red dashed lines in Supplementary Figure S1), seasonality makes less of a difference to the incidence in under-fives (Supplementary Figure S2c), except at high prevalences when seasonal variation results in lower incidence for a given prevalence.

Supplementary References

54 Bonnet, S. *et al.* Estimation of malaria transmission from humans to mosquitoes in two neighbouring villages in south Cameroon: evaluation and comparison of several indices. *Transactions of the Royal Society of Tropical Medicine and Hygiene* **97**, 53-59 (2003).

55 Boudin, C., Olivier, M., Molez, J.-F., Chiron, J.-P. & Ambroise-Thomas, P. High Human Malarial Infectivity to Laboratory-Bred Anopheles gambiae in a Village in Burkina Faso. *Am J Trop Med Hyg* **48**, 700-706 (1993).

56 Githeko, A. K. *et al.* The reservoir of Plasmodium falciparum malaria in a holoendemic area of western Kenya. *Transactions of the Royal Society of Tropical Medicine and Hygiene* **86**, 355-358 (1992).

57 Carnevale, P. P., Frézil, J. J. L., Bosseno, M. M. F., Le Pont, F. F. & Lancien, J. J. [The aggressiveness of Anopheles gambiae A in relation to the age and sex of the human subjects]. *Bulletin of the World Health Organization* **56**, 147-154 (1978).

58 Port, G. R., Boreham, P. F. L. & Bryan, J. H. The Relationship of Host Size to Feeding by Mosquitos of the Anopheles-Gambiae Giles Complex (Diptera, Culicidae). *Bulletin of Entomological Research* **70**, 133-144 (1980).

59 Smith, T., Charlwood, J. D., Takken, W., Tanner, M. & Spiegelhalter, D. J. Mapping the densities of malaria vectors within a single village. *Acta Tropica* **59**, 1-18 (1995).

60 Eyles, D. E. & Young, M. D. The duration of untreated or inadequately treated Plasmodium falciparum infections in the human host. *Journal* **10**, 327-336 (1951).

61 Falk, N. *et al.* Comparison of pcr-rflp and genescan-based genotyping for analyzing infection dynamics of plasmodium falciparum. *Am J Trop Med Hyg* **74**, 944-950 (2006).

62 Zwang, J. *et al.* Safety and Efficacy of Dihydroartemisinin-Piperaquine in Falciparum Malaria: A Prospective Multi-Centre Individual Patient Data Analysis. *PLoS ONE* **4**, e6358, doi:10.1371/journal.pone.0006358 (2009).

63 Miller, M. J. Observations on the natural history of malaria in the semi-resistant West African. *Transactions of the Royal Society of Tropical Medicine and Hygiene* **52**, 152-168 (1958).

64 Watkins, W. M., Mberu, E. K., Winstanley, P. A. & Plowe, C. V. The efficacy of antifolate antimalarial combinations in Africa: a predictive model based on pharmacodynamic and pharmacokinetic analyses. *Parasitology Today* **13**, 459-464 (1997).

65 Collins, W. E. & Jeffery, G. M. A retrospective examination of mosquito infection on humans infected with Plasmodium falciparum. *American Journal of Tropical Medicine and Hygiene* **68**, 366-371 (2003).

66 Dunyo, S. *et al.* Gametocytaemia after Drug Treatment of Asymptomatic Plasmodium falciparum. *PLOS Clin Trial* **1**, e20, doi:10.1371/journal.pctr.0010020 (2006).

67 Collins, W. E. & Jeffery, G. M. A retrospective examination of secondary sporozoite- and trophozoite-induced infections with Plasmodium falciparum: development of parasitologic and clinical immunity following secondary infection. *The American Journal of Tropical Medicine and Hygiene* **61**, 20-35 (1999).

68 Deloron, P. & Chougnet, C. Is Immunity to malaria really short-lived? *Parasitology Today* **8**, 375-378, doi:10.1016/0169-4758(92)90174-z (1992).

69 Sehgal, V. M., Siddiqui, W. A. & Alpers, M. P. A seroepidemiological study to evaluate the role of passive maternal immunity to malaria in infants. *Transactions of the Royal Society of Tropical Medicine and Hygiene* **83**, 105-106 (1989).

70 Snow, R. W., Menon, A. & Greenwood, B. M. Measuring morbidity from malaria. *Annals of Tropical Medicine and Parasitology* **83**, 321-323 (1989).

71 Abramowitz, M. & Stegun, I. A. Handbook of Mathematical Functions with Formulas, Graphs, and Mathematical Tables. National Bureau of Standards Applied Mathematics Series 55. Tenth Printing. (1972).
72 Cameron, A. C. & Trivedi, P. *Regression analysis of count data*. 1 edn, (Cambridge University Press, 1998).
73 Rapuoda, B. *et al.* The efficacy of antimalarial monotherapies, sulphadoxine-pyrimethamine and amodiaquine in East Africa: implications for sub-regional policy. *Tropical Medicine & International Health* **8**, 860-867 (2003).
